# Publisher Correction: R-loop formation and conformational activation mechanisms of Cas9

**DOI:** 10.1038/s41586-023-06779-x

**Published:** 2023-10-30

**Authors:** Martin Pacesa, Luuk Loeff, Irma Querques, Lena M. Muckenfuss, Marta Sawicka, Martin Jinek

**Affiliations:** Department of Biochemistry, University of Zurich, Zurich, Switzerland

**Keywords:** DNA, Genetic engineering, Cryoelectron microscopy, X-ray crystallography, RNA

Correction to: *Nature* 10.1038/s41586-022-05114-0 Published online 24 August 2022

In the version of the article initially published, there were errors in Fig. 1. In Fig. 1a and b, the dark blue strands, now labelled “NTS”, were mislabelled “TS”. In Fig. 1b the right panel was missing the label “8-nt match”, and Fig. 1c was missing the label “6-nt match”. These errors have been corrected in the HTML and PDF versions of the article.

