# Supplementary material for: R-loop formation and conformational activation mechanisms of Cas9
Source: Nature. 2022 Aug 24;609(7925):191–6. doi: 10.1038/s41586-022-05114-0 (PMC9433323; doi:10.1038/s41586-022-05114-0)
Supplement: Supplementary file 1 — Oligonucleotide sequences used in the study. [file 41586_2022_5114_MOESM1_ESM.docx]

**Supplementary Table 1 | Oligonucleotide sequences used in the study.**

PAM sequences underlined.

|  | **Sequence (5' - 3')** | **Purpose** |
| --- | --- | --- |
| 6-nt complementary DNA target for cryoEM (TS) | CGTGATTCCAGCGTCTTGAAGTCTACATAG | Target DNA strand of the 6-nt complementary substrate used to determine the 6-nt match complex using cryoEM. |
| 6-nt complementary DNA target for cryoEM (NTS) | CTATGTAGACTTCATATTATTGGAATCACG | Non-target DNA strand of the 6-nt complementary substrate used to determine the 6-nt match complex using cryoEM. |
| 8-nt complementary DNA target for cryoEM (TS) | CGTGATTCCAGCGTCTCAAAGTCTACATAG | Target DNA strand of the 8-nt complementary substrate used to determine the 8-nt match complex using cryoEM. |
| 8-nt complementary DNA target for cryoEM (NTS) | CTATGTAGACTTGCTATTATTGGAATCACG | Non-target DNA strand of the 8-nt complementary substrate used to determine the 8-nt match complex using cryoEM. |
| 10-nt complementary DNA target for x-ray (TS) | CGTGATTCCAGCGTCTCATCGTCTACATAG | Target DNA strand of the 10-nt complementary substrate used to determine the 10-nt match complex using crystallisation. |
| 10-nt complementary DNA target for x-ray (NTS) | CTATGTAGACACGCTATTATTGGAATCACG | Non-target DNA strand of the 10-nt complementary substrate used to determine the 10-nt match complex using crystallisation. |
| 10-nt complementary DNA target for cryoEM (TS) | CGTGATTCCAGCGTCTCATCAAATACGCAGCG | Target DNA strand of the 10-nt complementary substrate used to determine the 10-nt match complex using cryoEM. |
| 10-nt complementary DNA target for cryoEM (NTS) | CGCTGCGTATTTACGCTATTATTGGAATCACG | Non-target DNA strand of the 10-nt complementary substrate used to determine the 10-nt match complex using cryoEM. |
| 12-nt complementary DNA target for cryoEM (TS) | CGTGATTCCAGCGTCTCATCTTATACGCAGCG | Target DNA strand of the 12-nt complementary substrate used to determine the 12-nt match complex using cryoEM. |
| 12-nt complementary DNA target for cryoEM (NTS) | CGCTGCGTATGGACGCTATTATTGGAATCACG | Non-target DNA strand of the 12-nt complementary substrate used to determine the 12-nt match complex using cryoEM. |
| 14-nt complementary DNA target for cryoEM (TS) | CGTGATTCCAGCGTCTCATCTTTAACATAGCTCG | Target DNA strand of the 14-nt complementary substrate used to determine the 14-nt match complex using cryoEM. |
| 14-nt complementary DNA target for cryoEM (NTS) | CGAGCTATGTCGGGACGCTATTATTGGAATCACG | Non-target DNA strand of the 14-nt complementary substrate used to determine the 14-nt match complex using cryoEM. |
| 16-nt complementary DNA target for cryoEM (TS) | CGTGATTCCAGCGTCTCATCTTTATGATAGTACTCG | Target DNA strand of the 16-nt complementary substrate used to determine the 16-nt match complex using cryoEM. |
| 16-nt complementary DNA target for cryoEM (NTS) | CGAGTACTATTGCGGGACGCTATTATTGGAATCACG | Non-target DNA strand of the 16-nt complementary substrate used to determine the 16-nt match complex using cryoEM. |
| 18-nt complementary DNA target for cryoEM, checkpoint (TS) | CGTGATTCCAGCGTCTCATCTTTATGCGAGTGTACTCG | Target DNA strand of the 18-nt complementary substrate used to determine the inactive 18-nt match complex using cryoEM. |
| 18-nt complementary DNA target for cryoEM, checkpoint (NTS) | CGAGTACACTATTGCGGGACGCTATTATTGGAATCACG | Non-target DNA strand of the 18-nt complementary substrate used to determine the inactive 18-nt match complex using cryoEM. |
| 18-nt complementary DNA target for cryoEM, catalytically active (TS) | CGTGATTCCAGCGTCTCATCTTTATGCGAGTGTACTCGTCTTGC | Target DNA strand of the 18-nt complementary substrate used to determine the catalytically active 18-nt match complex using cryoEM. |
| 18-nt complementary DNA target for cryoEM, catalytically active (NTS) | GCAAGACGAGTACACTCGCATAAAACGCTATTATTGGAATCACG | Non-target DNA strand of the 18-nt complementary substrate used to determine the catalytically active 18-nt match complex using cryoEM. |
|  |  |  |
| λ1 sgRNA (2-stem loop) | GACGCAUAAAGAUGAGACGCGUUUUAGAGCUAGAAAUAGCAAGUUAAAAUAAGGCUAGUCCGUUAUCAACUUGAAAAAGUG | In vitro transcribed SpCas9 λ1 sgRNA with 2 stem loops for reconstitution of complexes used for structure determination using crystallisation. |
| λ1 sgRNA (3-stem loop) | GACGCAUAAAGAUGAGACGCGUUUUAGAGCUAGAAAUAGCAAGUUAAAAUAAGGCUAGUCCGUUAUCAACUUGAAAAAGUGGCACCGAGUCGGUGCUUUU | In vitro transcribed SpCas9 λ1 sgRNA with 3 stem loops for reconstitution of complexes used for structure determination using cryoEM. |
| TRAC sgRNA (3-stem loop) | AGAGUCUCUCAGCUGGUACAGUUUUAGAGCUAGAAAUAGCAAGUUAAAAUAAGGCUAGUCCGUUAUCAACUUGAAAAAGUGGCACCGAGUCGGUGCUUUU | In vitro transcribed SpCas9 TRAC sgRNA with 3 stem loops for biochemical cleavage assays. |
|  |  |  |
| TRAC on-target cleavage substrate (TS) | /Atto532/CGACAATACCGTGTACCAGCTGAGAGACTCTAGATGAGC | Atto532 labeled target DNA strand for in vitro cleavage of TRAC on-target substrate |
| TRAC on-target cleavage substrate (NTS) | GCTCATCTAGAGTCTCTCAGCTGGTACACGGTATTGTCG | Non-target DNA strand for in vitro cleavage of TRAC on-target substrate |
| TRAC off-target2 cleavage substrate (TS) | /Atto532/CGACAATACCATGTACCAGCTGTGTGACTTTAGATGAGC | Atto532 labeled target DNA strand for in vitro cleavage of TRAC off-target2 substrate |
| TRAC off-target2 cleavage substrate (NTS) | GCTCATCTAAAGTCACACAGCTGGTACATGGTATTGTCG | Non-target DNA strand for in vitro cleavage of TRAC off-target2 substrate |
| TRAC off-target4 cleavage substrate (TS) | /Atto532/CGACAATACCATGTACTAGCTGGGAGACTTCAGATGAGC | Atto532 labeled target DNA strand for in vitro cleavage of TRAC off-target4 substrate |
| TRAC off-target4 cleavage substrate (NTS) | GCTCATCTGAAGTCTCCCAGCTAGTACATGGTATTGTCG | Non-target DNA strand for in vitro cleavage of TRAC off-target4 substrate |
| TRAC off-target4-MismatchPosition5 cleavage substrate (TS) | /Atto532/CGACAATACCATGTATCAGCTGGGAGACTTCAGATGAGC | Atto532 labeled target DNA strand for in vitro cleavage of TRAC off-target4 substrate, with mismatched moved from position 6 to position |
| TRAC off-target4-MismatchPosition5 cleavage substrate (NTS) | GCTCATCTGAAGTCTCCCAGCTGATACATGGTATTGTCG | Non-target DNA strand for in vitro cleavage of TRAC off-target4 substrate, with mismatched moved from position 6 to position |
| TRAC off-target4-NoSeedMismatch cleavage substrate (TS) | /Atto532/CGACAATACCATGTACCAGCTGGGAGACTTCAGATGAGC | Atto532 labeled target DNA strand for in vitro cleavage of TRAC off-target4 substrate, with removed seed mismatch |
| TRAC off-target4-NoSeedMismatch cleavage substrate (NTS) | GCTCATCTGAAGTCTCCCAGCTGGTACATGGTATTGTCG | Non-target DNA strand for in vitro cleavage of TRAC off-target4 substrate, with removed seed mismatch |
| TRAC off-target5 cleavage substrate (TS) | /Atto532/CGACAATACCATGTAACAGCTGTGAGACTCCAGATGAGC | Atto532 labeled target DNA strand for in vitro cleavage of TRAC off-target5 substrate |
| TRAC off-target5 cleavage substrate (NTS) | GCTCATCTGGAGTCTCACAGCTGTTACATGGTATTGTCG | Non-target DNA strand for in vitro cleavage of TRAC off-target5 substrate |
